# Model-Informed Precision Dosing (MIPD)

**DOI:** 10.3390/pharmaceutics14122731

**Published:** 2022-12-06

**Authors:** Jonás Samuel Pérez-Blanco, José M. Lanao

**Affiliations:** 1 Area of Pharmacy and Pharmaceutical Technology, Department of Pharmaceutical Sciences, University of Salamanca, 37007 Salamanca, Spain; 2 The Institute for Biomedical Research of Salamanca (IBSAL), 37007 Salamanca, Spain

Model-informed precision dosing (MIPD) is an advanced quantitative approach focusing on individualized dosage optimization, integrating complex mathematical and statistical models of drugs and disease combined with individual demographic and clinical patient characteristics. MIPD has been highlighted as a current and useful tool for drug dosage optimization in drug development processes and clinical practice.

This Special Issue focuses on new MIPD strategies and methodologies to optimize drug dosage in specific populations. A total of twenty manuscripts, nineteen research articles and one review article, are published in this Special Issue.

Population pharmacokinetics (popPK) and dose optimization in different types of patients constitutes a useful working tool within the framework of the MIPD approach. Population pharmacokinetic models are of essential in the selection of doses of a wide variety of drugs and especially drugs with a narrow therapeutic margin in special populations, such as pediatric patients, critically ill patients, etc.

In this Special Issue, a total of eleven manuscripts on popPK and the dosage of various drugs are presented, addressing antibiotics, including linelozid, meropenem, imipenem and ciprofloxacin, in special populations such as critically ill patients. Other popPK studies included in this Special Issue focus on different drugs, such as valproic acid in pediatric and adult Caucasian patients, donezepil administered in patch formulations, levetiracetam in critically ill patients with normal or augmented renal function, and monoclonal antibodies such as infliximab or adalimumab for anti-TNF therapy in patients with inflammatory bowel disease.

One of the manuscripts focuses on building a popPK model to establish MIPD algorithm to optimize the dosing of linezolid in patients with multidrug and extensively drug-resistant tuberculosis and propose three sampling occasions to derive an individualized dose that results in effective and safe dosage regimens [1].

Considering the therapeutic importance of the use of antibiotics in patient populations with great inter- and intra-individual variability, this Special Issue includes MIPD-based dosing strategies for antibiotics, such as meropenem, imipenem and ciprofloxacin, for patients in intensive care units. Based on a popPK model of meropenem for critically ill adult patients using probability target attainment (PTA), prolonged infusion or a high-dosage regimen of meropenem is proposed, particularly when treating critically ill patients with increased renal clearance or those infected with pathogens with decreased in vitro susceptibility. This study also concludes that extracorporeal membrane oxygenation (ECMO) use does not affect meropenem PK in critically ill patients [2].

A tabular precision dosing tool for the initial therapy of meropenem, integrating hospital-specific pathogen susceptibility and based on popPK in intensive care units, was developed [3]. Parametric and non-parametric popPK models were also optimized for the dosage of imipenem in critically ill patients, which is appropriate for individuals with high glomerular filtration rates (eGFRs), but insufficient for low eGFRs [4].

In another comparative study, an individual and popPK analysis of ciprofloxacin in critically ill patients in the first 36 h of treatment was performed. No differences were reported between ciprofloxacin pharmacokinetic parameters between early and late phases of treatment, and creatinine clearance was identified as a covariate of ciprofloxacin pharmacokinetics [5]. In this same type of population, a study on the population pharmacokinetics of the antiepileptic levetiracetam in critically ill patients with normal or augmented renal function was carried out, proposing specific dosing schemes for this kind of population [6].

The good predictability of a popPK model of valproic acid was found based on the characterization of the valproic acid apparent clearance (CL/F) in pediatric and adult Caucasian patients. The popPK model demonstrates the influence of co-administration with carbamazepine, phenytoin, and phenobarbital on the pharmacokinetics of valproic acid [7].

A two-compartmental popPK model of the antipsychotic donezepil with two transit compartments was used for the characterization of this drug after the administration of transdermal patches in comparison with the oral route of administration, establishing dose equivalence between both routes of administration [8]. Additionally, and in the therapeutic group of antipsychotics, a review on the PK and pharmacogenetic data regarding the three major long-acting injectable (LAI) atypical antipsychotics, risperidone, paliperidone, and aripiprazole, is included in this Special Issue. The main conclusions of this review are the role of CYP2D6 in the PK of LAI aripiprazole and common aspects in the popPK models developed for these drugs, such as the influence of body weight, administration site, and needle characteristics. The manuscript also suggests that the combination of pharmacogenetics and PK leads to individualized dosing antipsychotic therapy [9].

This Special Issue also includes three papers on popPK and the dosing of two monoclonal antibodies, infliximab and adalimumab in patients with gastrointestinal tract diseases, such as ulcerative colitis and Crohn’s disease.

The predictive performance of twelve different published infliximab popPK models for inflammatory bowel disease patients was evaluated. Two of the models were suggested to have the best predictive performance and may be used as MIPD approaches for infliximab therapy in this kind of population [10]. A similar manuscript allows the best predictive performance for two Adalimumab popPK models to be established [11].

Different dosing strategies have been tested in comparison to standard dosing for increasing infliximab exposure during induction therapy in patients with ulcerative colitis. A dose of 10 mg/kg improves the probability of endoscopic improvement together with dose adaptation based on the interindividual variability of the patients [12].

In addition, this Special Issue includes several studies on pharmacokinetic/pharmacodynamic models (PK/PD). Focusing on applications in the field of infectious diseases, three manuscripts of this type are included. The anti-pseudomonal activity of different antibiotics against *P. aeruginosa* was evaluated using a PK/PD analysis with three different PK/PD indices based on the probability of target attainment (PTA). According to the results of this study, the most active antibacterial against *P. aeruginosa* was ceftazidime/avibactam, followed by ceftolozane/tazobactam and colistin [13]. In the same way, PK/PD analysis was used as a tool to optimize the treatment of *Neisseria gonorrhoeae* infections. The conclusions of this study suggest that ceftriaxone and oral cefixime are good candidates for treating gonorrhea [14].

A semi-mechanistic pharmacokinetic/pharmacodynamic (PK/PD) modelling and simulation approach was tested to evaluate the activity of Amphotericin B against *Candida auris* in vitro. The model includes two fungal stages consisting of a drug-susceptible fungal subpopulation and a drug-resistant subpopulation. In addition, a modified Emax sigmoidal model better describes this drug effect [15].

Neutropenia is usually associated with palbociclib toxicity in patients with breast cancer who are treated with this drug. A PK/PD model of five compartments, including a blood compartment, stem cell compartment and three transit compartments, was used to evaluate the relationship between plasma concentrations of palbociclib and absolute neutrophile count. According to the results, palbociclib < 100 µg/L can limit the risk of grade 4 neutropenia [16].

The dosing individualization of ustekimab in Crohn’s disease was based on a semi-mechanistic popPK/PD model composed of a two-compartment PK model linked to an indirect response model. This model allows individualized treatments with ustekimab in patients with Crohn´s disease [17].

Bayesian algorithms are a fundamental element of the MIPD framework because they can be used to forecast individualized dosing to obtain target therapeutic concentrations in patients enrolled in therapeutic drug monitoring (TDM) programs. In this Special Issue, several papers focus on this objective.

Bayesian software is an important tool in hospitals for the routine dosage individualization of a wide spectrum of drugs in different patient populations undergoing TDM programs. Posologyr is an open-source R package developed for Bayesian individual parameter estimation and dose individualization with different drugs. The performance of this computer program was tested against NONMEM for maximum a posteriori (MAP) points estimates and against Monolix for the estimation of full posterior distributions of individual parameters in a wide variety of models [18].

In this Special Issue, two articles focused on Bayesian forecasting for dosage adaptation following TDM. One of these addresses the predictive performance in vancomycin TDM testing five different model-based approaches and suggests the potential benefit of model-based vancomycin dosing in adult patients compared with the standard TDM [19]. The other contribution focuses on the use of the routine TDM of tyrosine kinase inhibitors (TKI), such as erlotinib, imatinib, lapatinib and sorafenib in cancer therapy. The results of the study demonstrate the high inter- and intra-individual variability in the PK behavior of this type of drug, as well as the utility of routine TDM to optimize doses and assess adherence, as well as interactions with food and other drugs [20].

In summary, this Special Issue demonstrates the rise of the MIPD as a powerful tool in the field of precision medicine for the optimization of treatments, with direct implications in increasing the safety and efficacy of pharmacological treatments.
